# Advancing biomedical engineering: Leveraging Hjorth features for electroencephalography signal analysis

**DOI:** 10.2478/joeb-2023-0009

**Published:** 2023-12-31

**Authors:** Wissam H. Alawee, Ali Basem, Luttfi A. Al-Haddad

**Affiliations:** 1 Control and Systems Engineering Department, University of Technology- Iraq, Baghdad, Iraq; 2 Training and Workshops Center, University of Technology- Iraq, Baghdad, Iraq; 3 Air Conditioning Engineering Department, Faculty of Engineering, Warith Al-Anbiyaa University, Karbala, Iraq

**Keywords:** Biomedical engineering, electroencephalography, Hjorth features, machine learning, signal processing

## Abstract

Biomedical engineering stands at the forefront of medical innovation, with electroencephalography (EEG) signal analysis providing critical insights into neural functions. This paper delves into the utilization of EEG signals within the MILimbEEG dataset to explore their potential for machine learning-based task recognition and diagnosis. Capturing the brain’s electrical activity through electrodes 1 to 16, the signals are recorded in the time-domain in microvolts. An advanced feature extraction methodology harnessing Hjorth Parameters—namely Activity, Mobility, and Complexity—is employed to analyze the acquired signals. Through correlation analysis and examination of clustering behaviors, the study presents a comprehensive discussion on the emergent patterns within the data. The findings underscore the potential of integrating these features into machine learning algorithms for enhanced diagnostic precision and task recognition in biomedical applications. This exploration paves the way for future research where such signal processing techniques could revolutionize the efficiency and accuracy of biomedical engineering diagnostics.

## Introduction

Biomedical engineering integrates principles from engineering with biological sciences to advance healthcare and medical practices [1–3]. Among the various tools at its disposal, electroencephalography (EEG) stands out due to its non-invasive measurement of brain activity, which are vital for diagnosing neurological states, understanding brain functions, and even developing brain-computer interfaces [4, 5].

EEG signals reflect the complex neurodynamics of the brain, with their analysis offering insights into normal and pathological states [6]. Traditionally, EEG analysis has relied on visual inspection and frequency-based approaches in addition to nonlinear features; however, these methods are often time-consuming and may lack the sensitivity needed for precise diagnosis required for advanced applications [7]. With the advent of machine learning and data-driven analytics in biomedical engineering, there is a growing interest in leveraging computational methods for EEG analysis [8, 9]. The application of these methods promises to enhance the recognition of patterns within EEG signals, leading to improved therapeutic strategies [10, 11].

This paper presents an exploration of EEG signal analysis using the MILimbEEG dataset [12, 13]. The focus lies on the extraction of Hjorth parameters—Activity, Mobility, and Complexity—from time-domain EEG signals recorded via electrodes [14]. These features encapsulate essential characteristics of signal variability, signal frequency, and signal complexity, respectively.

The significance of the Hjorth parameters extends beyond their mathematical definitions, offering a deeper understanding of the underlying brain dynamics [15, 16]. By presenting a correlation analysis and clustering behavior of these features, this study aims to demonstrate their potential in augmenting machine learning models for disease recognition and task classification.

As biomedical engineering continues to evolve, the integration of advanced signal processing techniques, as proposed in this paper, leads to novel discoveries in medical diagnostics and expands the horizons of neural engineering.

In structuring the paper, Section 2 explores the utilized dataset on EEG-based signals. The Hjorth parameters are exhibited in detail in Section 3. Section 4 gives a thorough analysis on the visual representation, correlation analysis, and clustering behavior of the methodology while explaining the potential of machine learning-based diagnosis. The paper concludes with section 5 highlighting the main achieved points with potential future work considerations.

## Materials and methods

At the heart of this investigation lies the MILimbEEG Dataset, an extensive collection of EEG recordings harvested from a heterogeneous group of 60 individuals, which includes both scholarly participants and clinical patients as referenced in [12, 13]. This rich dataset encompasses a total of 8680 EEG recordings, each spanning a duration of four seconds, meticulously gathered, and formatted into CSV files for analytical convenience. The contributions from each participant are substantial, with 124 individual files recorded per experimental session. This volume of data ensures a dataset of significant depth and variety, positioning it as an excellent candidate for machine learning analyses that demand substantial and varied training inputs.

**Fig.1:**
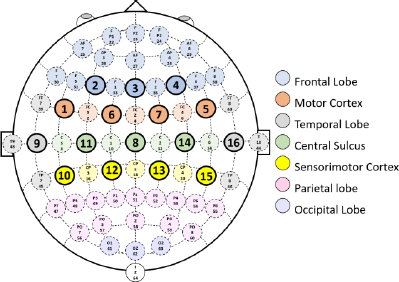
A schematic representation of the brain regions selected for EEG signal recording [13].

**Fig.2:**
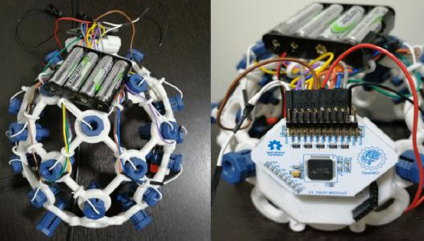
The OpenBCI Cyton with Daisy Biosensing Board and associated EEG headgear, detailing the hardware setup used in signal collection [13].

**Fig.3:**
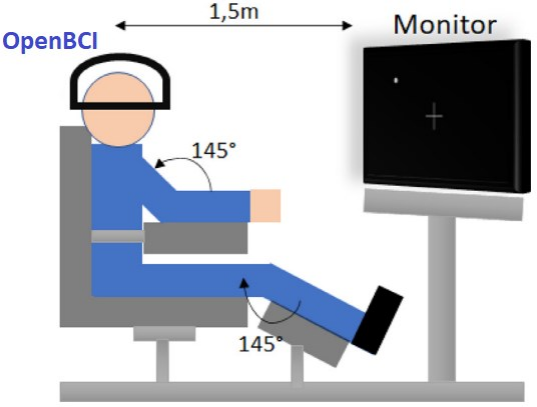
The reclining chair with delineated limb angles and the monitor placement at a 1.5-meter distance, ensuring direct alignment with the participant’s field of vision [13].

Figure 1 depicts the specific cerebral locales from which these signals were acquired, presenting the targeted Brodmann areas within the context of the internationally recognized 10-10 system for electrode placement. Additionally, Figure 2 showcases the OpenBCI Cyton and Daisy Biosensing Board, along with the specialized EEG headgear employed for capturing the EEG signals. Moreover, the design of the experimental framework is carefully crafted to ensure the precision and trustworthiness of the data. Subjects are comfortably positioned in a specially designed reclining chair that supports the body’s natural posture, with limbs arranged at specific angles. Visual prompts for the tasks are projected onto a screen positioned at a distance of 1.5 meters and aligned with the participants’ line of sight to facilitate ease of interaction. Figure 3 provides a graphical depiction of the data collection setup.

The EEG data acquisition is carried out using the OpenBCI Cyton and Daisy Biosensing Board, which sample at 125 Hz and apply a bandpass filter with cutoffs at 5 and 50 Hz to ensure data quality. The configuration includes 16 non-invasive dry electrodes, supplemented by two reference electrodes for grounding, to comprehensively record the electrical activity of the brain. The recorded data is divided into segments corresponding to various tasks involving hand and foot movements, in addition to intervals of inactivity. This segmentation results in a multifaceted dataset, providing a rich resource for the complex pattern recognition capabilities of advanced machine learning algorithms.

The EEG recordings obtained from the experiments act as critical markers of neuronal activity in response to discrete limb movements. Each four-second segment captured provides insight into the cerebral processes associated with either the physical enactment or the cognitive rehearsal of a given task. Table 1 summarizes the spectrum of participant actions cataloged in the study, encompassing motions of both hands and feet, along with instances of rest. These tasks will be presented from a selected subject to progress through the improved Hjorth features to understand their structure and potential for machine learning classification.

**Tab.1:** Task abbreviations assigned to different limb movements.

Task	Description
BEO	Recording a Baseline with Eyes Open
CLH	Closing Left Hand: five times per run
CRH	Closing Right Hand: five times per run
DLF	Dorsal flexion of Left Foot: five times per run
PLF	Plantar flexion of Left Foot: five times per run
DRF	Dorsal flexion of Right Foot: five times per run
PRF	Plantar flexion of Right Foot: five times per run
Rest	Resting in between tasks: after each task

## Hjorth Feature Extraction

Hjorth parameters provide a time-domain analysis method for EEG signals, capturing essential features of signal power, frequency content, and complexity. They are particularly useful for acquiring the statistical properties of EEG signals in studies related to cognitive and neurological tasks. A description of each parameter is provided below.

### Activity

The Activity parameter is the mean square value of the amplitude, representing the power of the EEG signal. It is calculated using equation 1 [16]:

(1) $$Activity\;=Var(x)=\frac{1}{N}\sum_{i=1}^{N}{\left(X_{i}-AM_{x}\right)}^{2}$$

where *X_i_* is the signal amplitude at the *i^th^* sample, *AM_x_* is the mean amplitude of the signal over N samples, *N* is the total number of samples. This parameter is indicative of the signal variance and is a direct measure of the signal’s power.

### Mobility

Mobility is a measure of the mean frequency or the rate of change in the signal, providing an estimate of the signal’s standard deviation. It is obtained from the variance of the first derivative of the signal as follows: First, the difference between consecutive signal amplitudes is calculated in equation 2 below:

(2) $$d_{i}=x_{i+1}-x_{i}$$

Then, the variance of these differences is calculated using equation 3 [17]:

(3) $$Var(d)=\frac{1}{N-1}\sum_{i=1}^{N}{\left(d_{i}-AM_{d}\right)}^{2}$$

Where *d_i_* is the first derivative at the *i^th^* sample, *AM_d_* is the mean value of *d* over *N* samples. The Mobility is then computed as the square root of the ratio of the variance of the first derivative of the signal to that of the signal itself as written in equation 4 [18]:

(4) $$Mobility\;=\sqrt{\frac{Var(d)}{Var(x)}}$$

This parameter reflects the frequency content of the signal by measuring the standard deviation of the power spectrum.

### Complexity

Complexity compares the signal’s pattern to the pattern of a pure sine wave, indicating the signal’s pattern changes. It is calculated as the Mobility of the first derivative of the signal divided by the Mobility of the signal as equation 5 states [19]:

(5) $$Complexity\;=\frac{\;Mobility\;of\;d}{\;Mobility\;of\;x}$$

This dimensionless metric is indicative of the signal’s complexity, with higher values suggesting a more complex signal structure compared to a simple sine wave.

By integrating these parameters into the analysis, a rich feature set is derived that is capable of improving the performance of machine learning models in classifying and interpreting EEG signals, as will be stated in the following context.

## Results and discussion

This section of the study is crucial since it gives the results of applying Hjorth parameters to EEG data and explores their significance in the context of biomedical engineering and machine learning. The study is divided into three sections: an evaluation of the time-domain signals, a comprehensive examination of the extracted features, and an exploration of prospective improvements via machine learning processing.

### Time-domain signals

The time-domain analysis of EEG signals provides the initial layer of understanding, showcasing the fluctuations of neural activities. Here, the section details the observed patterns within the EEG signals as they correspond to different limb movements and rest periods, establishing a baseline for the feature extraction process.

**Fig.4:**
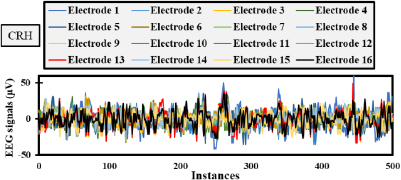
EEG signals over a 4-second interval with 500 instances for Subject 10 in CRH phase.

**Fig.5:**
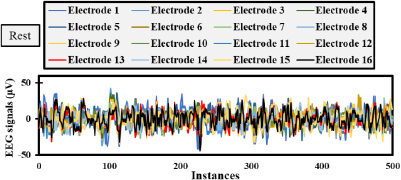
EEG signals over a 4-second interval with 500 instances for Subject 10 in Resting phase.

Randomly selected, Figures 4 and 5 depict the electrode responses in microvolts for an interval of 500 instances for two tasks, namely CRH and Resting, respectively. More intense fluctuations are noticed when analyzing Figure 4 due to the presence of a movement while slightly less amplitudes or fluctuations are observed in the resting task due to the absence of any movement by the subject. These values are chosen randomly to subject 10 and the features will be calculated to the same individual to understand the potential of these same patterns.

### Extracted features analysis

Moving deeper into the data, we dissect the Hjorth parameters extracted from the time-domain signals. The statistical significance of Activity, Mobility, and Complexity is evaluated to discern how these features can be correlated with specific motor tasks or mental states, revealing distinct behavioral clusters within the dataset.

**Fig.6:**
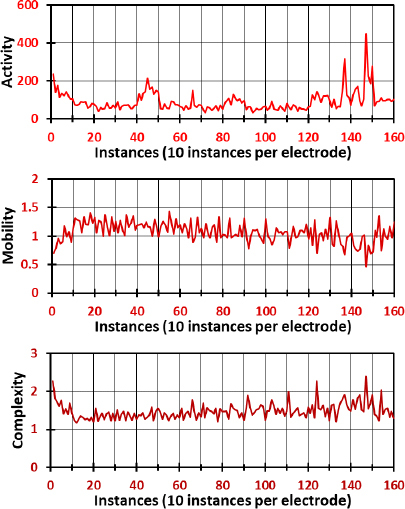
Hjorth parameters of the Resting movement, namely activity, mobility, and complexity for all electrodes.

In Figure 6, the Hjorth parameters for the Resting movement across all electrodes are depicted through a tripartite series of sub-figures. Each of these sub-figures is dedicated to one of the three dynamic statistical measures: activity, mobility, and complexity. These parameters provide insight into the signal’s variance, the frequency of the signal, and the similarity of the signal’s waveform to a pure sine wave, respectively. Notably, electrodes 14 and 15 exhibit markedly high amplitudes across all Hjorth parameters, suggesting a pronounced signal variability, frequency content, and waveform complexity at these locations. This could be indicative of localized neural dynamics or a heightened responsiveness to the resting state, to describe the unique patterns of neural activity captured by electrodes 14 and 15. Conversely, electrodes 7 and 13 are characterized by high fluctuation trends across all parameters, which may reflect a different neural mechanism or a response to physiological variables not captured by the other electrodes. The observed patterns in activity, mobility, and complexity could have significant implications for understanding the resting state’s neural underpinnings and call for a more nuanced analysis of electrode-specific activity.

**Fig.7:**
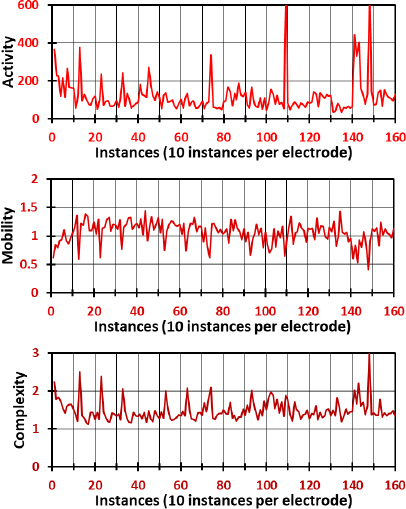
Hjorth parameters of the CRH movement, namely activity, mobility, and complexity for all electrodes.

Figure 7 delineates the Hjorth parameters of the CRH movement, where the data present a stark contrast to the resting state. This figure illustrates an overall increase in fluctuation and amplitude across all electrodes. The activity parameter values range broadly from 0 to 600, indicating a wide variance in signal power during the CRH movement, which may be associated with the diverse motor and cognitive demands of this task. Mobility values extend from 0.3 to 1.4, suggesting variability in the frequency content of the signals that could be reflective of the complex motor control strategies employed during CRH movement. Complexity values between 1.1 and 3 indicate a range of waveform shapes, from simple oscillatory patterns to more irregular and complex forms. These variations can be attributed to the intricate neural coordination required for the CRH movement, highlighting the sophisticated interplay between different brain regions during this task.

A comparison between Figures 6 and 7 reveals distinct patterns of neural activity associated with the different movements. While high amplitude in Hjorth parameters in the Resting movement is localized mainly to electrodes 14 and 15, the CRH movement is characterized by a universal elevation in fluctuation and amplitude across all parameters and electrodes. This suggests that the CRH movement engages a more extensive network of brain regions than the resting state, leading to increased variance, frequency content, and waveform complexity. The differential patterns observed in activity, mobility, and complexity between the two figures underscore the dynamism of the brain’s response to varying cognitive and motor demands. The marked contrasts between the resting and CRH states may provide valuable insights into the neural correlates of task-specific activities and the adaptability of the neural networks involved. These distinctions could pave the way for further research into the electrophysiological signatures of different brain states and movements, potentially contributing to the development of targeted interventions or diagnostic criteria for neurological conditions.

**Fig.8:**
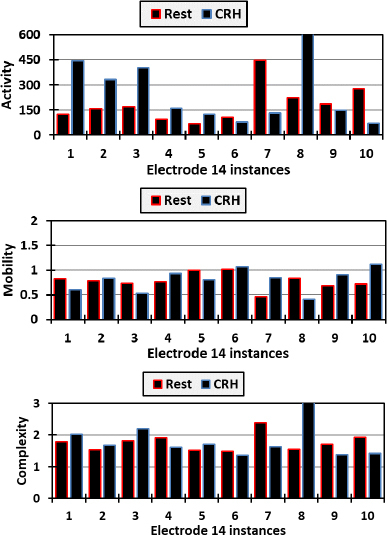
Activity, mobility, and complexity of electrode 14 readings for both resting and closing right hand.

**Fig.9:**
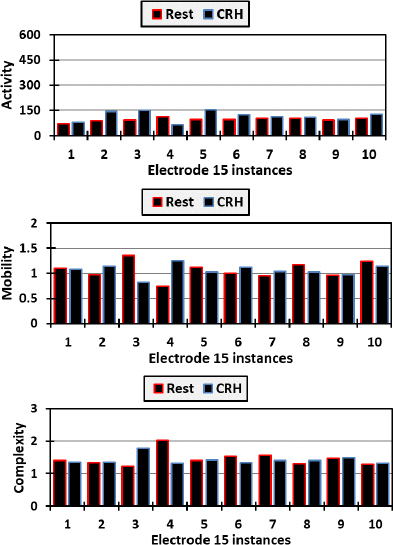
Activity, mobility, and complexity of electrode 15 readings for both resting and closing right hand.

Figure 8 elucidates the behavior of Hjorth parameters for electrode 14 during Resting and CRH tasks, revealing distinct patterns of neural activity. During the Resting task, activity levels varied substantially, with a peak value suggesting intermittent spikes in neural activity, which could be indicative of spontaneous brain activity or external noise. Mobility remained relatively stable, suggesting a consistent frequency change, while complexity fluctuated, pointing to varying degrees of signal irregularity, perhaps reflecting the natural ebb and flow of resting brain dynamics. In contrast, the CRH task manifested higher overall activity levels with a broader range, implying increased neural engagement necessary for motor function. Mobility displayed greater variation, indicating more dynamic changes in frequency content, which aligns with the complex coordination required for hand movement. Complexity reached higher levels compared to the Resting task, suggesting a more intricate neural pattern during active movement. These fluctuations highlight electrode 15’s potential proximity to brain regions responsible for motor control, underscoring its significance in studies of neural dynamics and motor activity. To elaborate more, Figure 9 presents the instances of electrode 15 as well in comparison with those of figure 8.

### Machine Learning Progressing

Finally, the extracted features serve as inputs to machine learning models, aiming to classify tasks and diagnose conditions with greater accuracy. We discuss the integration of these features into various algorithmic frameworks, their impact on model performance, and the prospects they hold for the future of automated EEG analysis and biomedical applications.

Machine learning models have increasingly incorporated statistical features from EEG data to enhance classification and diagnostic capabilities. Deep Neural Networks (DNN) [20, 21], with their capacity for feature learning, have shown promise in deciphering complex patterns from Hjorth parameters and similar statistics. The hierarchical nature of DNNs allows them to distill high-level abstractions from raw data, which is particularly beneficial for identifying subtle neurological differences between various cognitive tasks or pathological states. Stacking models [22, 23], which combine the predictions of multiple machine learning algorithms to produce an ensemble, have also been utilized to capitalize on the strengths of individual models. This approach can yield more robust predictions, as it leverages the diverse perspectives of different models. Stochastic Gradient Descent (SGD) [24, 25], a cornerstone of many learning algorithms due to its efficiency in handling large datasets, has been adapted for EEG data to optimize performance in real-time applications, enhancing the potential for on-the-fly analyses in clinical settings.

Looking forward, the integration of statistical EEG features into machine learning models holds substantial promise for advancing the field of biomedical engineering. The next frontier includes refining these models to cope with the high dimensionality and variability inherent in EEG data. For instance, previous works have achieved a high classification accuracy while advancing machine learning-based algorithms with Hjorth parameters to optimize the models in the domain of EEG signals [26, 27]. As computational power and algorithmic sophistication advance, there is potential for developing real-time diagnostic systems and personalized medicine applications. This could lead to breakthroughs in understanding and treating neurological conditions, as well as in deploying brain-computer interfaces (BCIs) with unprecedented precision. Moreover, the fusion of EEG data with other biomarkers and the application of transfer learning to leverage pre-trained models on extensive datasets may unlock new pathways for comprehensive, cross-modal diagnostics. The continuous evolution of machine learning methodologies is poised to play a pivotal role in the translation of EEG analysis from research laboratories to clinical practice, heralding a new era of data-driven neuroscience.

## Conclusion

In conclusion, this paper embarked on a comprehensive journey through the intricate landscape of EEG signal analysis, focusing on the extraction and elucidation of Hjorth parameters as pivotal statistical features. It meticulously detailed the methodologies involved in preprocessing EEG signals, extracting critical features, and employing these features as inputs for advanced machine learning models. The research presented a thorough examination of the Hjorth parameters—activity, mobility, and complexity—across different electrodes and movements, providing a granular analysis of their behavior and implications in the context of EEG data interpretation.

The findings of this paper shed light on the distinctive patterns and fluctuations of Hjorth parameters that are indicative of various neurological states and movements. By analyzing these parameters in resting and movement-associated tasks, the study unearthed significant variations, which were then adeptly correlated with specific electrodes. For future work, the machine learning section of the study leveraged good insights to future developments of enhancing the accuracy of task classification and condition diagnosis, offering a promising outlook and methodology for the application of such methodologies in automated EEG analysis. The paper concluded with a discussion on the impact of these features on machine learning model performance and pondered the future prospects of this approach, potentially revolutionizing the fields of biomedical engineering, neurology, and beyond.
